# Effects of Employment Status and Motivations on the Onset of Social Isolation in Old Age: A 2.5-year Longitudinal Study

**DOI:** 10.31662/jmaj.2025-0006

**Published:** 2025-08-01

**Authors:** Hiroshi Murayama, Yoko Muto, Mai Takase, Isuzu Nakamoto, Kumiko Nonaka, Yoshinori Fujiwara

**Affiliations:** 1 Research Team for Social Participation and Healthy Aging, Tokyo Metropolitan Institute for Geriatrics and Gerontology, Tokyo, Japan; 2 The Tokyo Metropolitan Support Center for Preventative Long-term and Frail Elderly Care, Tokyo Metropolitan Institute for Geriatrics and Gerontology, Tokyo, Japan; 3 Tokyo Metropolitan Institute for Geriatrics and Gerontology, Tokyo, Japan

**Keywords:** employment, motivation, social isolation, old age, Japan

## Abstract

**Introduction:**

Although prior research has investigated the health benefits of paid work in old age, its effect on social isolation, a crucial societal issue, remains unclear. Moreover, not only employment status but also the motivation for working may play a vital role in understanding social isolation. This study aimed to investigate the effect of employment status and motivations on the onset of social isolation in older Japanese people.

**Methods:**

Longitudinal data were collected from self-administered questionnaire surveys of residents aged 65 and older dwelling in an area in Ota Ward in the Tokyo Metropolitan area in 2015 (baseline) and 2018 (follow-up). This study included 1,556 participants who were not socially isolated at baseline. Social isolation was defined as face-to-face or non-face-to-face interactions occurring less than once per week. Employment status was categorized as having or not having paid work, whereas motivations for working were classified as “financial reasons only,” “non-financial reasons only (such as health, *ikigai*, social contribution, and social connection),” or “both financial and non-financial reasons.”

**Results:**

Among the total participants (men: 38.2%, average age: 72.9 years), 36.1% were employed at baseline, and 20.1% became socially isolated at follow-up. A binary logistic regression analysis with the adjustment for potential covariates showed no significant association between employment status and the onset of social isolation. However, individuals who worked only for financial reasons were more likely to experience social isolation than were those who did not work or worked for non-financial reasons.

**Conclusions:**

Although employment status was not directly associated with social isolation, the motivations for working influenced its onset. Non-financial motivations for work in later life may reduce the risk of social isolation.

## Introduction

Social participation is a key determinant of successful and healthy aging, with employment serving as an important form of social participation. In Japan, the most rapidly aging society globally, approximately 30% of the population aged 65 years and older, and a quarter of the population older than 65 years engages in paid work ^(1)^. Employment in old age is associated with significant health benefits. Systematic reviews have shown that older adults who work have a reduced risk of all-cause mortality ^(2)^, cognitive decline ^(3)^, and poor self-rated health ^(4)^. Furthermore, individual studies revealed the positive effects of employment on functional disability ^(5)^ and mental health and well-being ^(6), (7), (8)^.

Why do older adults work? According to the Japanese government’s report on the country’s aging population, motivations for working include income, health, social connections, and social contribution ^(9)^. Because motivations vary among individuals, understanding the reasons for working is crucial for designing effective job-seeking support and promoting sustained employment among older adults. Notably, the positive effect of working varies according to the reasons for working. Nemoto et al. ^(10)^ reported that older people who only worked for financial reasons experience poorer self-rated health and greater functional decline than do those who work for meaningful purposes, such as *ikigai* (finding a sense of purpose in life).

Another critical focus of this study is social isolation. Social isolation is a crucial determinant of health in older adults, given several studies have reported that reduced social connections are associated with adverse health outcomes^(11), (12)^. Although life expectancy has been increasing in Japan, social isolation has become a severe social issue, exacerbated by the growing number of single-person and older adult-only households, and of those with individuals living with dementia.

Various interventions have been developed to mitigate social isolation in older adults^(13), (14)^, and encouraging participation in social activities is a key strategy. In particular, employment may help prevent social isolation because it is a way to connect with society ^(15)^. However, few studies have evaluated the relationship between employment status in old age, including the reasons for working, and social isolation.

Given this context, this study aimed to evaluate the effects of employment status and motivations for working on the onset of social isolation, using longitudinal data from older Japanese adults living in the community. As mentioned above, employment could contribute to better health conditions ^(2), (3), (4), (5), (6), (7), (8)^. Moreover, working for financial motivation was associated with poorer health status than working for non-financial motivation ^(10)^. Social isolation could occur owing to health issues ^(16), (17)^. Therefore, we hypothesized that 1) those who were currently employed were less likely to be socially isolated than were those who were unemployed, and 2) those who worked for non-financial reasons were less likely to experience social isolation than were those who worked for financial reasons.

## Materials and Methods

### Study participants

We used data from a 2.5-year longitudinal, self-administered questionnaire survey of older residents living in Iriarai area in Ota Ward in the Tokyo Metropolitan area, Japan. Baseline and follow-up surveys were conducted in August 2015 and January 2018, respectively. As of April 2015, the total population of Ota Ward was 709,907, with 22.4% aged 65 years and older.

**Figure 1.**
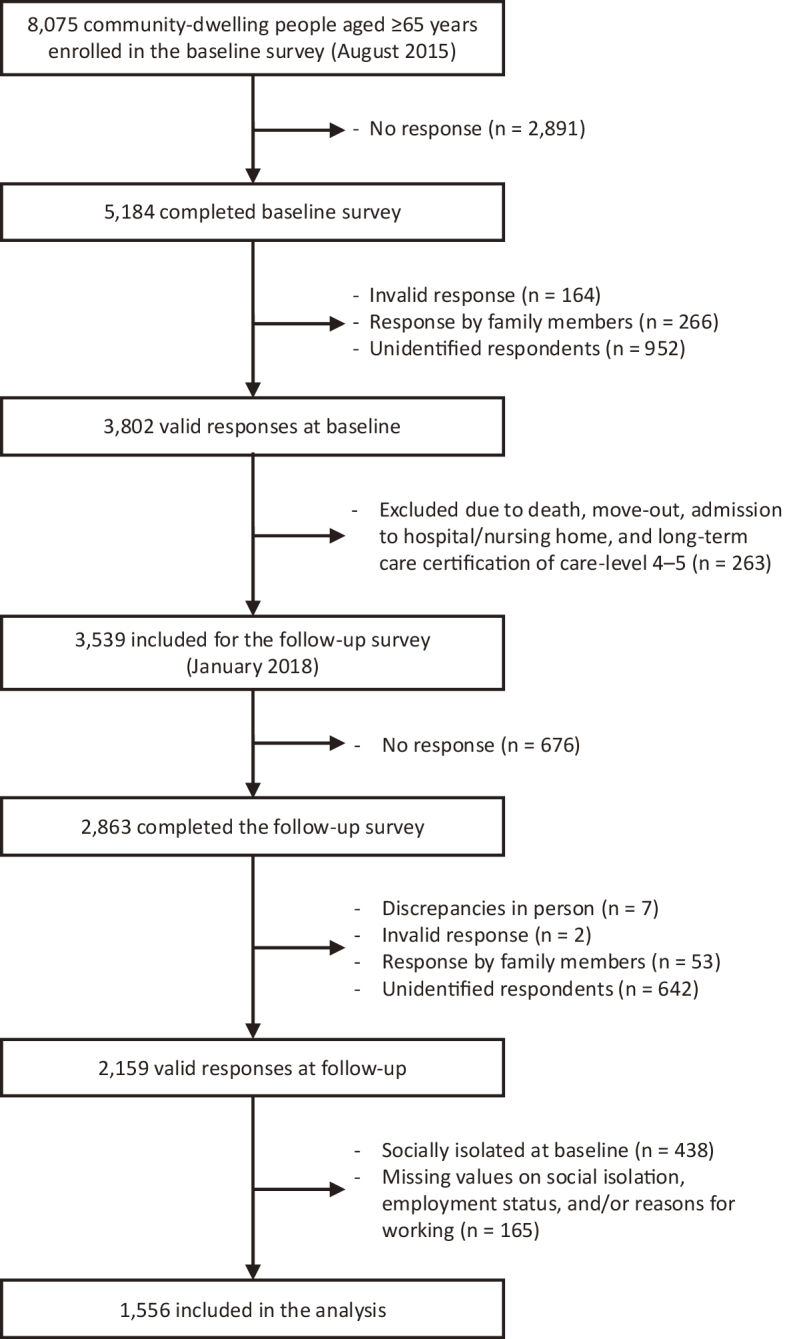
Flow diagram of study participants.

Figure 1 presents a flow diagram of the survey participants. The target sample at baseline included all residents aged 65 years and older in Iriarai area, Ota Ward, excluding those living in nursing homes, hospitalized, or classified as care-level 4-5 in long-term care insurance. A total of 8,075 people were enrolled in the baseline survey, 5,184 of whom returned the questionnaire (response rate: 64.2%). Among these questionnaires, 3,802 responses were deemed valid. In the follow-up survey, we excluded those who had died, moved, been hospitalized, entered nursing homes, or were classified as care-level 4-5 in long-term care insurance after the baseline survey, and 3,539 responses were included. We obtained 2,863 responses, and after excluding 704 invalid responses, 2,159 respondents who answered both baseline and follow-up surveys were identified (follow-up rate: 61.0%). Because this study focused on the effect of employment on social isolation, 438 socially isolated individuals at baseline were excluded, along with 165 participants with missing data on social isolation, employment status, and/or working reasons at baseline. As a result, the final sample comprised 1,556 observations.

The study protocol was approved by the ethics committee of the Tokyo Metropolitan Institute for Geriatrics and Gerontology (approved on April 23, 2015 [approval number: 23-164]). All questionnaires included a written explanation of the purpose of the survey and stated that participation was voluntary. Responding to the questionnaire was deemed consent to participate.

### Social isolation

Social isolation was defined as having fewer than weekly face-to-face or non-face-to-face interactions with relatives and friends, excluding cohabiting family members^(18), (19)^. To assess interaction frequency, participants were asked four questions: 1) How often do you meet or go out with friends or neighbors?; 2) How often do you talk on the phone with friends and neighbors (including through e-mail and fax)?; 3) How often do you see or go out with family members or relatives you do not live with?; and 4) How often do you talk on the phone with family members or relatives you do not live with? The possible answers were: 6-7 times a week (almost every day), 4-5 times a week, 2-3 times a week, once a week, 2-3 times a month, once a month, less than once a month, or never. Following previous studies^(18), (19)^, the total interaction frequency was calculated, with interactions occurring less than once a week classified as “social isolation.”

### Employment status and reasons for working

Employment status was assessed with a single item: “Do you currently have paid work (including part-time work or help in the family business)?” Respondents answered with one of the following four options: 1) working more than 35 hours per week, 2) working fewer than 35 hours per week or irregularly, 3) not working but looking for work, or 4) not working or not looking for work. Participants were categorized as “working” (1 and 2) or “not working” (3 and 4).

For those categorized as “working,” we asked about employment type (“self-employed,” “employee,” or “other”), weekly working hours, and years in the current occupation. In addition, according to the earlier study ^(10)^, the following reasons for working were assessed: 1) to earn money for living, 2) to repay debts, 3) to earn pocket money, 4) to maintain health, 5) to gain a sense of purpose in life, 6) to contribute to and make a connection with society, 7) to make good use of time, 8) following recommendations from family or others, and 9) others. Respondents could select multiple reasons. We defined reasons 1-3 as “financial motivation” and 4-9 as “non-financial motivation.” Participants were further categorized into three groups based on their reasons for working: “financial reasons only (FR),” “non-financial reasons only (NFR),” or “both financial and non-financial reasons (BR).”

### Covariates

Demographic factors included sex (“men” or “women”), age (in years), marital status (“married” or “unmarried”), household composition (“living alone” or “cohabiting”), and length of residence in Ota Ward (“< 30 years,” “30-49 years,” or “≥ 50 years”). Socioeconomic status was measured by educational years (“6-9 years,” “10-12 years,” or “≥ 13 years”) and subjective financial status (“affluent,” “neither,” or “poor”). Health-related factors included comorbidities, self-rated health (“good” or “poor”), and instrumental activities of daily living (IADL) scores. Regarding the comorbidities, we calculated the number of chronic diseases (hypertension, heart disease, cerebrovascular disease, cancer, and diabetes mellitus) and divided them into three categories accordingly (“0,” “1,” or “≥ 2”). The IADL was assessed using a subscale of the Tokyo Metropolitan Institute of Gerontology Index of Competence ^(20)^, with scores ranging from 0 to 5, whereby higher scores indicate greater independence. Social factors included frequency of community activity participation (“non-participation,” “less than once a week,” or “once a week and over”) and frequency of going out per month (times).

### Statistical analysis

First, we described participants’ baseline characteristics by employment status and reasons for working. Second, we performed binary logistic regression analysis to assess the effects of employment status and reasons for working on social isolation for the total sample, using non-working as the reference category. We controlled for demographic factors, socioeconomic status, health-related factors, and social factors. Third, focusing on those working at baseline, we conducted a binary logistic regression analysis to examine the differential effect of the reasons for working on social isolation, controlling for employment type, working hours per week, and years in the current occupation, in addition to the above covariates. We used two modeling strategies: one controlling for sex and age (model 1) and the other adjusting for the covariates in addition to sex and age (model 2). The results are presented as odds ratios (ORs) and 95% confidence intervals (CIs). Statistical analysis was performed using IBM SPSS Statistics version 23 (IBM, Armonk, NY, USA).

## Results

Table 1 lists the baseline characteristics of participants. Men accounted for 38.2% of the sample, with an average age of 72.9 (standard deviation: 5.8), and 36.4% (n = 562) of the sampled individuals worked. Working people were mostly men, younger, and married, and had better health conditions than did non-working people. Among working people, 23.3% (n = 131), 36.1% (n = 203), and 40.6% (n = 228) worked for FR, NFR, and BR, respectively. The proportion of women was lower in people working for only NFR, and their mean age was older than the other groups. They also had higher education and more affluent financial status. In contrast, those who worked for only FR had lower education and poorer financial status. They also had longer working hours. A total of 20.1% became socially isolated in the follow-up survey, with the highest proportion comprising those working for FR.

**Table 1.** Participants’ Characteristics at Baseline.

	All (N = 1,556)	Working (n = 562)			Not working (n = 994)
		Working for FR (n = 131)	Working for NFR (n = 203)	Working for BR (n = 228)	
Sex (%)
Men	38.2	56.5	43.3	50.9	31.8
Age (years; mean ± SD)	72.9 ± 5.8	70.7 ± 4.7	72.2 ± 5.2	70.6 ± 5.4	73.8 ± 6.0
Marital status (%)
Married	64.1	63.3	70.6	66.8	62.3
Household composition (%)
Living alone	23.0	27.0	19.5	22.6	23.3
Duration of residence (%)
< 30 years	25.6	28.2	24.4	24.2	25.8
30-49 years	36.8	29.8	39.3	41.4	36.1
≥ 50 years	37.7	42.0	36.3	34.4	38.1
Educational year (%)
6-9 years	9.7	16.3	6.5	8.5	9.8
10-12 years	41.3	34.9	36.7	44.4	42.4
≥ 13 years	49.0	48.8	56.8	47.1	47.8
Subjective financial status (%)
Affluent	42.5	29.5	66.7	42.1	39.4
Neither	38.2	34.9	26.3	33.9	42.0
Poor	19.3	35.7	7.1	24.0	18.6
Comorbidities (%)
0	37.5	34.6	32.9	40.1	38.2
1	45.6	50.0	43.5	43.1	46.0
≥ 2	16.9	15.4	23.5	16.8	15.8
Self-rated health (%)
Good	85.1	88.0	89.0	93.2	82.0
IADL score (ranging from 0 to 5; mean ± SD)	4.9 ± 0.4	5.0 ± 0.3	5.0 ± 0.3	4.9 ± 0.3	4.9 ± 0.4
Frequency of community activity participation (%)
Non-participation	28.5	33.6	28.6	28.1	27.9
< once a week	31.5	37.4	27.6	34.2	30.9
≥ once a week	40.0	29.0	43.8	37.7	41.2
Frequency of going out per month (times; mean ± SD)	29.5 ± 17.5	29.9 ± 20.2	31.8 ± 19.2	31.7 ± 18.6	28.3 ± 16.4
Employment type (%)^a^
Self-employed	37.2	45.4	40.9	29.2	-
Employed	48.6	46.2	34.8	61.9	-
Other	14.3	8.5	24.2	8.8	-
Working hours (%)^a^
≥ 35 hours per week	42.2	48.9	40.4	39.9	-
Years of current occupation^a^ (mean ± SD)	23.1 ± 18.3	24.2 ± 18.2	26.0 ± 17.9	19.7 ± 18.3	-
Changes in social isolation status from baseline to follow-up (%)
Changed (socially isolated at the follow-up)	20.1	29.2	14.6	23.7	19.2

BR: both financial and non-financial reasons; FR: financial reasons; IADL: instrumental activities of daily living; NFR: non-financial reasons; SD: standard deviation. ^a^Values were calculated among participants working at baseline.

Table 2 presents the association between baseline employment status and social isolation at follow-up. After controlling for covariates, we observed no significant association between working status at baseline and the onset of social isolation (model 2: OR = 1.12, 95% CI = 0.81-1.55). Table 3 presents the association between the reasons for working and social isolation, setting non-working as a reference. The FR group was more likely to be socially isolated at follow-up than were non-working individuals (model 2: OR = 1.90, 95% CI = 1.15-3.14), whereas the other two groups (i.e., NFR and BR) did not have significant associations with social isolation.

**Table 2.** Association between Employment Status and the Onset of Social Isolation.

	Model 1	Model 2
	OR (95% CI)	OR (95% CI)
Employment status (ref: not working)
Working	1.01 (0.77-1.33)	1.12 (0.81-1.55)
Sex (ref: women)
Men	2.38 (1.85-3.09)	2.25 (1.62-3.13)
Age (every 1-year increase)	1.01 (0.98-1.03)	1.00 (0.97-1.03)
Marital status (ref: unmarried)
Married		1.00 (0.64-1.55)
Household composition (ref: living alone)
Cohabiting		1.41 (0.86-2.34)
Duration of residence (ref: < 30 years)
30–49 years		0.74 (0.50-1.09)
≥ 50 years		1.12 (0.77-1.63)
Educational year (ref: ≥ 13 years)
6–9 years		1.34 (0.97-1.85)
10–12 years		1.14 (0.68-1.91)
Subjective financial status (ref: affluent)
Neither		1.10 (0.78-1.56)
Poor		1.31 (0.87-1.96)
Comorbidities (ref: 0)
1		1.02 (0.73-1.42)
≥ 2		0.95 (0.60-1.49)
Self-rated health (ref: good)
Poor		1.22 (0.82-1.82)
IADL scores (every 1-point increase)		0.93 (0.65-1.35)
Frequency of community activity participation (ref: non-participation)
< once a week		0.45 (0.32-0.65)
≥ once a week		0.39 (0.27-0.56)
Frequency of going out per month (every 1-time increase)		1.00 (0.99-1.01)

CI: confidence interval; IADL: instrumental activities of daily living; OR: odds ratio; ref: reference.

**Table 3.** Association between Reasons for Working and the Onset of Social Isolation in the Total Sample.

	Model 1	Model 2
	OR (95% CI)	OR (95% CI)
Reasons for working (ref: not working)		
Working for FR	1.47 (0.96-2.26)	1.90 (1.15-3.14)
Working for NFR	0.65 (0.42-1.00)	0.70 (0.42-1.17)
Working for BR	1.13 (0.78-1.64)	1.16 (0.75-1.77)
Sex (ref: women)		
Men	2.34 (1.80-3.04)	2.12 (1.52-2.97)
Age (every 1-year increase)	1.01 (0.98-1.03)	1.01 (0.98-1.03)
Marital status (ref: unmarried)		
Married		1.03 (0.66-1.61)
Household composition (ref: living alone)		
Cohabiting		1.41 (0.86-2.34)
Duration of residence (ref: < 30 years)		
30-49 years		0.74 (0.50-1.09)
≥ 50 years		1.10 (0.75-1.60)
Educational year (ref: ≥ 13 years)		
6–9 years		1.33 (0.96-1.85)
10–12 years		1.10 (0.75-1.60)
Subjective financial status (ref: affluent)		
Neither		1.02 (0.72-1.46)
Poor		1.18 (0.78-1.79)
Comorbidities (ref: 0)		
1		1.03 (0.74-1.44)
≥ 2		0.99 (0.63-1.57)
Self-rated health (ref: good)		
Poor		1.22 (0.82-1.83)
IADL scores (every 1-point increase)		0.93 (0.64-1.35)
Frequency of community activity participation (ref: non-participation)		
< once a week		0.45 (0.31-0.65)
≥ once a week		0.39 (0.27-0.57)
Frequency of going out per month (every 1-time increase)		1.00 (0.99-1.01)

BR: both financial and non-financial reasons; CI: confidence interval; FR: financial reasons; IADL: instrumental activities of daily living; NFR: non-financial reasons; OR: odds ratio; ref: reference.

Finally, Table 4 presents the association between the reasons for working and social isolation among people who worked at baseline. Older adults who worked for FR had a significantly higher likelihood of social isolation than did those who work for NFR (model 2: OR = 3.65, 95% CI = 1.75-7.61). However, there was no significant association between working for BR and social isolation in model 2.

**Table 4.** Association between Reasons for Working and the Onset of Social Isolation among the Sample Working at Baseline.

	Model 1	Model 2
	OR (95% CI)	OR (95% CI)
Reasons for working (ref: working for NFR)		
Working for FR	2.22 (1.26-3.89)	3.65 (1.75-7.61)
Working for BR	1.70 (1.01-2.86)	1.82 (0.94-3.52)
Sex (ref: women)		
Men	2.15 (1.39-3.30)	1.69 (0.93-3.10)
Age (every 1-year increase)	0.99 (0.95-1.03)	0.97 (0.92-1.03)
Marital status (ref: unmarried)		
Married		0.59 (0.28-1.25)
Household composition (ref: living alone)		
Cohabiting		1.93 (0.85-4.40)
Duration of residence (ref: < 30 years)		
30–49 years		0.88 (0.46-1.68)
≥ 50 years		1.23 (0.63-2.39)
Educational year (ref: ≥ 13 years)		
6–9 years		1.26 (0.72-2.19)
10–12 years		1.20 (0.50-2.89)
Subjective financial status (ref: affluent)		
Neither		0.54 (0.29-1.03)
Poor		0.78 (0.39-1.57)
Comorbidities (ref: 0)		
1		0.99 (0.57-1.73)
≥ 2		1.29 (0.61-2.72)
Self-rated health (ref: good)		
Poor		1.32 (0.59-2.96)
IADL scores (every 1-point increase)		0.88 (0.37-2.06)
Frequency of community activity participation (ref: non-participation)		
< once a week		0.58 (0.31-1.08)
≥ once a week		0.57 (0.30-1.06)
Frequency of going out per month (every 1-time increase)		1.00 (0.98-1.01)
Employment type (ref: self-employed)		
Employed		1.47 (0.75-2.88)
Other		1.49 (0.65-3.44)
Working hours (ref: ≥ 35 hours per week)		
< 35 hours per week		0.83 (0.46-1.50)
Years of current occupation (every 1-year increase)		1.00 (0.98-1.01)

BR: both financial and non-financial reasons; CI: confidence interval; FR: financial reasons; IADL: instrumental activities of daily living; NFR: non-financial reasons; OR: odds ratio; ref: reference.

## Discussion

This study evaluated the association between employment status/reasons for working and the onset of social isolation using longitudinal data from older people dwelling in the community in Japan. The results showed no association between employment status and the onset of social isolation. Although old-age employment is beneficial for peoples’ health, various working styles in old age could affect health conditions ^(8), (21), (22)^. There are more variations in working style in old age than in younger generations. For example, the proportion of older adults with full-time work is much lower ^(23)^. Therefore, the effect of employment status on the onset of social isolation might differ depending on the working style; thus, the association between working status and social isolation was not observed.

The onset of social isolation was found to vary by the motivations for working. In Table 3, people who worked for NFR were less likely to be socially isolated than were those who did not work, although not statistically significant (OR = 0.70 in model 2). This result shows that working for NFR, such as health, *ikigai*, social contribution, and social connection with society, could be a good prescription for social isolation prevention. Moreover, in Table 4, those who worked for BR (i.e., both FR and NFR) exhibited a smaller risk of social isolation than did those who worked for FR (OR = 1.82 for BR and 3.65 for FR in model 2). This implied that working for NFR could mitigate the risk of social isolation caused by working for FR.

Previous studies showed that social isolation in old age was predicted by sex (male), older age, living with others, poor self-rated physical and mental health, low frequency of going out, and non-participation in community activities ^(16), (17)^. In addition to these predictors, this study suggests the importance of focusing on the reasons for working among older working people. We assumed that people who worked only for FR might not have enough time to build/enjoy social relations with others and participate in social activities, such as hobbies and sports, causing social isolation. Individuals working for FR tended to participate less in social activities and work longer hours (more than 35 hours) than did other groups (i.e., NFR and BR; Table 1). However, although model 2 controlled for participation in community activities and working hours, the results remained unchanged. Hence, working for FR might influence social isolation, independent of these factors. A decrease in health conditions could be another possible explanation, given it was reported as a risk factor for social isolation ^(16), (17)^. A previous study reported that those only working for FR were more likely to have a decrease in their functional capacity than were those working for NFR ^(10)^. This phenomenon might lead to social isolation.

Other possible mechanisms would be through social relationships in the workplace and daily mental distress. With economic motivation being the primary driver, the desire for interaction and cooperation with others tends to be lower, leading to weak social connections in the workplace. As a result, they are more likely to be socially isolated. In addition, individuals who have financial worries tend to experience high levels of daily mental distress ^(24)^, which possibly leads to their social isolation status ^(16), (17)^. Moreover, a meta-analysis reported that those with low socioeconomic status such as earning low income were likely to have low self-esteem ^(25)^. This could affect their motivation and confidence in their work. These factors combined can make it harder for older adults to find fulfillment in their jobs, potentially leading to social isolation. Further research is warranted to elucidate this mechanism and to explore other mechanisms.

This study has some limitations. First, social isolation was defined as the frequency of face-to-face and non-face-to-face interactions with family members living apart, neighbors, and friends; however, interactions with colleagues in the workplace were not considered. In addition, this study did not consider the quality of relationships, but it might affect the results. Future studies should include these factors in the definition of social isolation. Second, although employment status is a time-varying factor, we did not consider the time-varying nature of employment status. Although some would continue the same jobs, others might quit or change the job at follow-up. Some who did not work at baseline might start working. This is the same case in the reasons for working. Third, because we excluded people who responded to the baseline survey only, there may be a selection bias. For example, people who responded to the baseline survey only tended to be older and less educated and have worse health conditions than did those who completed both surveys, and to be socially isolated (Supplementary Table 1). The results should be carefully interpreted. Fourth, there remain some confounding factors. For example, whether the respondent has a job that involves a lot of communication with others or a job that does not require communication would have some influence on the results. Workplace location (e.g., near/far from the respondents’ home) and a feeling toward work (e.g., job satisfaction) would also be important. Furthermore, although this study used subjective financial status as an indicator of socioeconomic status, it would be theoretically and practically important to exclude the possibility that motivations for working serve as a proxy for objective socioeconomic status, such as household income or financial assets. These should be considered in future research. Finally, the survey was conducted in a single urban area; therefore, the generalizability of this study’s results is somewhat limited.

In conclusion, although working was not associated with the onset of social isolation, the motivations for working are significantly associated with the risk of social isolation. Working for FR increases the risk of social isolation; thus, working for NFR may mitigate this risk. Globally, the number of people who work in later life has been increasing, and working in old age can be considered an opportunity to promote health and prevent social isolation. Given working for FR tends to induce social isolation in older people, assessing and considering their reasons for working is vital when supporting them in job-seeking and continuous employment.

## Article Information

### Conflicts of Interest

None

### Author Contributions

Hiroshi Murayama, Kumiko Nonaka, and Yoshinori Fujiwara designed the study and collected data. Hiroshi Murayama, Yoko Muto, Mai Takase, and Isuzu Nakamoto conducted data analyses. Hiroshi Murayama and Yoko Muto prepared the manuscript, and Mai Takase, Isuzu Nakamoto, Kumiko Nonaka, and Yoshinori Fujiwara provided critical feedback. All the authors have read and approved the final version of the manuscript.

### ORCiD iD

Hiroshi Murayama: https://orcid.org/0000-0003-2991-7763

### Approval by Institutional Review Board (IRB)

The study protocol was approved by the ethics committee of the Tokyo Metropolitan Institute for Geriatrics and Gerontology (approved on April 23, 2015 [approval number: 23-164]).

## Supplement

Supplementary Table 1
