# Supplementary material for: Effects of Employment Status and Motivations on the Onset of Social Isolation in Old Age: A 2.5-year Longitudinal Study
Source: JMA J. 2025 Aug 1;8(4):1098–107. doi: 10.31662/jmaj.2025-0006 (PMC12598144; doi:10.31662/jmaj.2025-0006)
Supplement: Supplementary Table 1 [file 2433-3298-8-4-1098-s001.pdf]

Supplementary Table 1. Differences in characteristics between people who participated in baseline survey and those who participated in both baseline and follow-up surveys

	People who participated in baseline survey only (n=1,643)	People who participated in both baseline and follow-up surveys (n=2,159)	p-value
Sex (%)			
Men	44.7	40.9	0.018[a]
Age (years old; mean ± SD)	75.3 ± 6.1	73.3 ± 7.1	<0.001[b]
Marital status (%)			
Married	58.3	63.0	0.003[a]
Household composition (%)			
Living alone	26.4	23.0	0.014[a]
Duration of residence (%)			<0.360[c]
<30 years	26.6	25.5	
30–49 years	32.5	36.7	
≥50 years	40.9	37.8	
Educational year (%)			<0.001[c]
6–9 years	19.2	12.3	
10–12 years	42.4	42.0	
≥13 years	38.4	45.7	
Subjective financial status (%)			<0.001[c]
Affluent	34.5	38.6	
Neither	28.1	22.4	
Poor	37.4	39.0	
Comorbidities (%)			0.003[c]
0	31.9	36.3	
1	46.7	45.4	
≥2	21.4	18.3	
Self-rated health (%)			
Good	72.3	81.8	<0.001[a]

IADL score (ranging from 0–5; mean ± SD)	4.7 ± 0.8	4.9 ± 0.5	<0.001[b]
Frequency of community activity participation (%)			<0.001[c]
Non-participation	47.1	35.9	
<once a week	28.5	30.6	
≥once a week	24.4	33.5	
Frequency of going out per month (times; mean ± SD)	26.2 ± 17.9	28.2 ± 17.5	<0.001[b]
Employment status (%)			
Working	64.6	64.8	0.934[a]
Social isolation status (%)			
Socially isolated	25.4	21.0	0.002[a]

IADL, instrumental activities of daily living; SD, standard deviation.

a: chi-squared test. b: t-test. c: Mann–Whitney U-test.
